# Recent increase in N_2_O growth rate (2013–2023) mainly due to increase of nitrogen-fertiliser and manure use in the Northern Tropics and Southern Landmass

**DOI:** 10.1186/s40562-026-00476-z

**Published:** 2026-04-28

**Authors:** Prabir K. Patra, Yasunori Tohjima, Akihiko Ito, Naveen Chandra, Motoki Sasakawa, Xin Lan, Bradley D. Hall, Paul B. Krummel, Ray F. Weiss, Christina M. Harth, Shinya Takatsuji, Daisuke Goto, Kumiko Takata, Luke M. Western, Ronald G. Prinn

**Affiliations:** 1 Research Institute for Global Change, JAMSTEC, Yokohama, 236-0001 Japan; 2 Seto Inland Sea Carbon Neutral Research Center, Hiroshima University, Hiroshima, 739-8529 Japan; 3 Research Institute for Humanity and Nature (RIHN), Kyoto, 603-8047 Japan; 4 Earth System Division, National Institute for Environmental Studies (NIES), Tsukuba, 305-8506 Japan; 5 Graduate School of Agricultural and Life Sciences, The University of Tokyo, Tokyo, 113-8657 Japan; 6 Global Monitoring Laboratory, National Oceanic & Atmospheric Administration (NOAA), Boulder, CO 80305 USA; 7 Cooperative Institute for Research in Environmental Sciences, University of Colorado Boulder, Boulder, CO 80309 USA; 8 CSIRO Environment, Aspendale, VIC 3195 Australia; 9 Scripps Institution of Oceanography, University of California, San Diego, CA 92093 USA; 10 Atmospheric Environment and Ocean Division, Atmosphere and Ocean Department, Japan Meteorological Agency (JMA), Tokyo, 105-8431 Japan; 11 National Institute of Polar Research, Tachikawa, 190-8518 Tokyo Japan; 12 Center for Sustainability Science and Strategy, Massachusetts Institute of Technology, Cambridge, MA 02139 USA; 13 Azabu University, Sagamihara, 252-5201 Japan

**Keywords:** Nitrous oxide, N_2_O emissions, Chemistry-transport model, Regional source-sink inversion, Nitrogen fertiliser, Natural soil, Oceanic exchange, Ecosystem nitrogen dynamics

## Abstract

Nitrous oxide (N_2_O) is a strong greenhouse gas that contributes significantly to global warming and causes depletion of ozone in the stratosphere. Recent observational records show an unprecedented acceleration in atmospheric N₂O growth, reaching 1.15 ppb yr^− 1^ in 2019–2023, a significant increase compared to 0.68 ppb yr^− 1^ in 2001–2005. This surge in growth rate is particularly pronounced over tropical regions, and has been measured most prominently at the southern-most island of Japan (Hateruma). In this study, we use N_2_O observations from globally distributed multi-institutional networks and the MIROC4-ACTM inversion framework to quantify N_2_O emissions and identify key regions that are driving the recent acceleration. Our results suggest that the major Asian countries, Brazil, Central and Northern Africa, and the Contiguous United States have increased emission sources in the recent 2.5 decades (1998–2023). Further, there has been an increase in land N_2_O emissions, at a rate of 106 GgN yr^− 1^ per year during 1998–2002 to 2019–2023 (1Gg = 10^9^g). The inversion inferred trends are consistent with increased fertiliser use and manure production to support extensive agriculture, and terrestrial ecosystem model results. The emissions from oceanic regions did not show significant increases in N_2_O (rate: 7 ± 2 GgN yr^− 1^ per year) in our inverse model setup. Our results underscore the importance for improved climate mitigation strategies and emissions reduction policies by increasing nitrogen-fertiliser use efficiency in agricultural land.

**Supplementary Information:**

The online version contains supplementary material available at 10.1186/s40562-026-00476-z.

## Introduction

Nitrous oxide (N₂O) is a long-lived greenhouse gas (GHG) with an atmospheric lifetime of approximately 109 ± 10 years and global warming potential of 273 at 100 year time horizon (Forster et al. 2021). Since preindustrial times, its concentration has risen from ~ 270 parts per billion (ppb) in 1750 to 336.69 ± 0.03 ppb in 2023, contributing significantly to both climate change and stratospheric ozone depletion (Rubino et al. 2019; Lan et al. 2025; Canadell et al. 2021; WMO/SAOD 2022). The current rate of increase is estimated at ~ 3.3% per decade during 2014–2023 (Prinn et al. 2018; Lan et al. 2025), driven primarily by microbial nitrification and denitrification in terrestrial and aquatic ecosystems. These microbial processes are influenced by environmental and biological factors, including soil moisture, temperature, oxygen availability, soil acidity, and nitrogen substrate levels (Porporato et al. 2003; Butterbach-Bahl et al. 2013; Bouwman et al. 2013; Tian et al. 2024; Zhu et al. 2025a, b). Anthropogenic activities—such as synthetic fertilizer use, livestock manure management, industrial emissions, and waste treatment—contribute substantially to the global N₂O budget (Thompson et al. 2019; Crippa et al. 2024; Ito et al. 2018), with emissions expected to rise in response to increasing global demands for food, feed, fiber and energy as per the assessment of the United Nations (UNEP/FAO 2024; Tubiello et al. 2022; Cui et al. 2021) and under climate change (Harris et al. 2022; Li et al. 2024; Vitousek et al. 1997).

Global N₂O emissions have increased from about 10.5 TgN yr^− 1^ (1 Tg = 10^12^ g) in the preindustrial era to ~ 17.0 TgN yr^− 1^ in the decade 2007–2016, with agriculture as the dominant driver (Prather et al. 2015; Canadell et al. 2021). Agricultural N_2_O emissions increased from 0.3 to 1.0 TgN yr^− 1^ in 1861 to 3.9–5.3 TgN yr^− 1^ by 2015, as estimated by the N_2_O Model Intercomparison Project (NMIP) (Tian et al. 2018). Given the urgency of limiting non-CO₂ GHG emissions to achieve climate targets, effective N_2_O mitigation strategies must reduce emissions without compromising food security. This underscores the importance of enhancing nitrogen use efficiency (NUE)—optimizing the fraction of nitrogen uptake by crops relative to nitrogen inputs—and adopting sustainable agricultural practices. Therefore, continuous monitoring of atmospheric N_2_O growth and identifying key drivers are critical for informed mitigation policies. Further large gaps persist among the estimated emissions of N_2_O by the terrestrial ecosystem models, inverse modelling of atmospheric data and the national reports (Del Grosso et al. 2022; Tian et al. 2024).

Recent observational records reveal an acceleration in atmospheric N_2_O growth rates since 2000, increasing from 0.68 ppb yr^− 1^ (2001–2005) to 0.98 ppb yr^− 1^ (2010–2015), reaching about 1.15 ppb yr^− 1^ in the most recent years (2019–2023) (Lan et al. 2025). This recent surge is particularly pronounced over tropical regions, marking an unprecedented rise in the past two decades and suggesting the agricultural practices and NUE dynamics may have changed regionally. In this study, we utilize global N₂O observation networks and the MIROC4-ACTM inversion framework at JAMSTEC (Patra et al. 2022) to quantify N₂O emissions and identify key regions driving the recent surge in atmospheric N₂O levels. This inverse model is one of the 4 models currently providing emission estimations to the Global N₂O Budget (Tian et al. 2024; Thompson et al. 2019).

## Methods

### Atmospheric observations

**Fig. 1:**
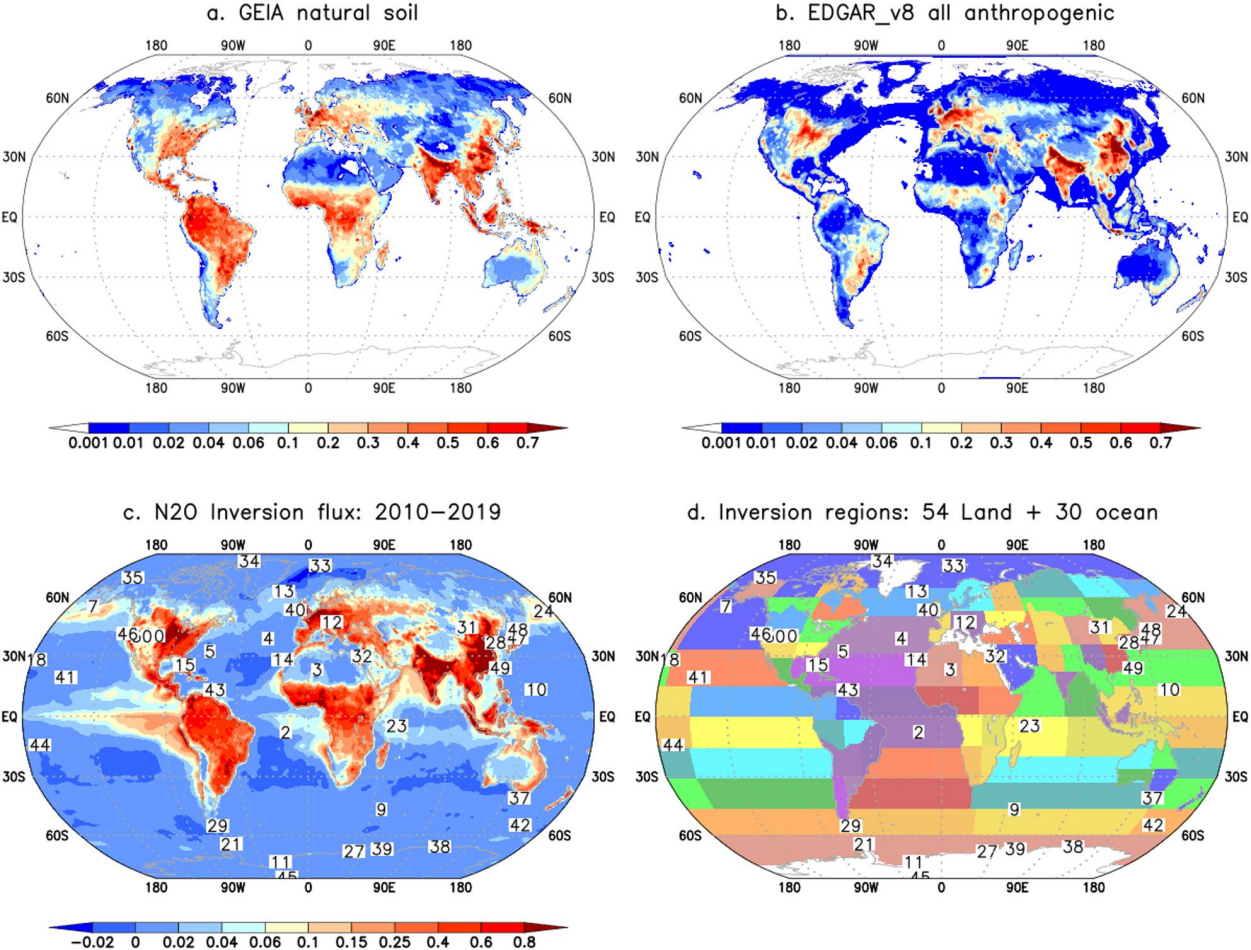
Maps of the Global Emission Initiative (GEIA) natural soil emissions (**a**), annual mean emissions from industrial and agricultural activities from the Emissions Database for Global Atmospheric Research, version 8 (EDGAR_v8) for 2010–2019 (**b**), total N_2_O fluxes estimated by MIROC-ACTM inverse model averaged over 2010–2019 (**c**). Location of 49 measurement sites (marked by the numbers) used in the inversion are shown on top of 84 inverse model regions (**d**). The inverse model has 30 regions over the ocean and 54 regions over the land (Patra et al. 2022)

We use observations from the National Oceanic & Atmospheric Administration (NOAA), Advanced Global Atmospheric Gases Experiment (AGAGE), Commonwealth Scientific and Industrial Research Organisation (CSIRO), National Institute for Environmental Studies (NIES), and Japan Meteorological Agency (JMA) (Huang et al. 2008; Prinn et al. 2018; Lan et al. 2024; Tojima et al. 2020; Takatsuji 2025). Some of the 49 sites have overlapping measurements from more than one institution (Table S1; Fig. 1d), with the number of unique sites at 40. Data from the NOAA, CSIRO and JMA sites are reported on the NOAA-2006 A calibration scale (also referred to as WMO_N2O_X2006A), while the NIES and AGAGE data are reported on the independent scales of NIES and SIO-2016, respectively (Hall et al. 2007). Co-located measurements from AGAGE (SIO-2016) and NOAA (NOAA-2006 A) at five common sites show site-dependent and time-dependent differences, amounting to ~ 0.5 ppb and ~ 0.2 ppb in the late 1990s and early 2020s, respectively. To account for the scale differences, we subtracted a fixed value of 0.5 ppb from the AGAGE data. We added 0.6 ppb to the NIES data, consistent with a -0.6 ppb offset relative to NOAA identified in WMO/IAEA Round Robin Comparison #6 (2014–2015) (https://gml.noaa.gov/ccgg/wmorr/wmorr_results.php?rr=rr6&param=n2o; last accessed 02 September 2025). We performed sensitivity inversion experiments to assess the time-dependent SIO-2016 vs. NOAA-2006 A scale difference, NOAA/AGAGE = 4.3641 × 10^− 5^ * Year + 0.91065 (Fig. S1).

Most of the measurement sites are located away from the emission hotspots seen in Fig. 1c but due to coarse transport model simulations at about 2.8^o^ × 2.8^o^ horizontal resolution (details in Sect.  2.2), simulations for some of the sites are affected by emission hotspots. Due to the presence of multiple sites in such cases, e.g., at TAP (site#28 in Fig. 1) in East Asia, and by assigning lower weightage of the sites (greater data uncertainty), the effect of single sites has been found to be negligible on inversion analyses. Similar to Patra et al. (2022), all the time series, observations and model simulations for the sites, are passed through a Butterworth bandpass filter with cutoff length of 24 months and fitted with 6 harmonics before using them in the inversion system (following Nakazawa et al. 1997).

### Atmospheric N_2_O forward and inverse models

The setups of our forward chemistry and transport for N_2_O simulation by MIROC4-ACTM have been discussed in Patra et al. (2018, 2024), and thus only a brief description is given here. The atmospheric general circulation model MIROC, version 4 is developed by the University of Tokyo, NIES and JAMSTEC (Watanabe et al. 2010). The MIROC4-ACTM meteorology is nudged toward the JMA’s 55-year Reanalysis (JRA-55; Kobayashi et al. 2015). We use MIROC4-ACTM at T42 horizontal resolution (42 spectral truncation) and 67-layer hybrid vertical coordinates. The lifetime of N_2_O in MIROC4-ACTM is about 127 years, which is in the range of the estimations by various assessment reports (Prather et al. 2015; Canadell et al. 2021; Burkholder and Hodnebrog 2023). The model transport properties in representing the Brewer-Dobson circulation and stratosphere-stratosphere exchange processes in MIROC4-ACTM using JRA-55 meteorology have been tested using multiple chemical tracers (Patra et al. 2018; Bisht et al. 2021).

We optimise fluxes from 84 partitions of the globe, with 54 over the land and 30 over the ocean surfaces, using a Bayesian inversion system (Patra et al. 2022). The flux corrections are then distributed on to a 1 × 1^o^ latitude-longitude grid based on the basis functions of 84 regions and regionally aggregated fluxes for 15 land regions (revised from 19 regions of Tian et al. 2024) and 11 ocean regions defined by Transcom 3 intercomparison project (Gurney et al. 2000) for analysis of the flux time series. Because the MIROC4-ACTM is run at about 2.8 ^o^×2.8^o^ horizontal resolution and our inversion is solving emissions for only 54 regions of the global land, we will only discuss emissions after aggregating the gridded emission data to 15 subcontinental scale land regions. The oceanic 30-region inversion fluxes are briefly discussed after aggregating to 11 regions.

The following equations are used to predict optimised (inversion) sources (S) and associated source error covariance matrices (C_S_) constrained by observation data (D_Obs_) and model simulation (D_ACTM_) with regional prior mean fluxes (S_0_) and prior flux error covariance matrix (C_S0_):

1 $$ {\mathrm{S}} = {\mathrm{S}}_{0} + \left( {{\mathrm{G}}_{{\mathrm{T}}} {\mathrm{C}}_{{\mathrm{D}}} ^{{ - {\mathrm{1}}}} {\mathrm{G}} + {\mathrm{C}}_{{{\mathrm{S}}0}} ^{{ - {\mathrm{1}}}} } \right)^{{ - {\mathrm{1}}}} {\mathrm{G}}^{{\mathrm{T}}} {\mathrm{C}}_{{\mathrm{D}}} ^{{ - {\mathrm{1}}}} \left( {{\mathrm{D}}_{{{\mathrm{Obs}}}} - {\text{ D}}_{{{\mathrm{ACTM}}}} } \right) $$

2 $$ {\mathrm{C}}_{{\mathrm{S}}} = \left( {{\mathrm{G}}_{{\mathrm{T}}} {\mathrm{C}}_{{\mathrm{D}}} ^{{ - {\mathrm{1}}}} {\mathrm{G}} + {\mathrm{C}}_{{{\mathrm{S}}0}} ^{{ - {\mathrm{1}}}} } \right)^{{ - {\mathrm{1}}}} $$

C_D_ is the measurement data error covariance matrix. The regionally uncorrelated prior flux uncertainties and measurement data uncertainties are calculated as the square root of C_S0_ and C_D_, respectively, and are represented by diagonal matrices. The C_S0_ are defined as 50% of regional total prior fluxes, and C_D_ are defined as (0.1 + RSD*0.3) ppb^2^ (following the best case identified in Patra et al. 2022). Site specific residual standard deviations (RSD) are calculated using the curve fitting to the model simulations (Nakazawa et al. 1997). Sites close to the high emission regions received lesser weightage due to greater RSD, as mentioned in Sect.  2.1.

### Prior emission cases

Inversion model estimated fluxes have large dependency on the prior flux emission distribution, particularly when the measurement network is not sufficiently dense (Fig. 1d) to capture the spatio-temporal variabilities in regional fluxes (Fig. 1c). Six different emission cases (Table 1) are formulated to test the impact of land and ocean fluxes on N_2_O inversion results based on the principles described in Patra et al. (2022). The oceanic prior fluxes are available as monthly-mean and repeating between years (referred to as cyclostationary) (Yang et al. 2020) but scaled for global totals to 3.2, 3.6 and 4.2 TgN yr^− 1^. Three ocean cases are prepared to account for prior flux selection on inversion results as reflected through the 6 inversion cases. Annual mean emissions from industrial activities are taken from Emissions Database for Global Atmospheric Research, version 8 (EDGAR_v8), including sectors of fossil fuel usage, chemical industry, industrial operations, transportation systems, waste management, biomass burning (Crippa et al. 2024). Natural soil emissions are taken from a fixed annual map from Global Emissions Inventory Activity (GEIA; Bouwman et al. 2013) and the Vegetation Integrative SImulator for Trace gases (VISIT; Ito et al. 2018). Agricultural soil emissions are also taken from EDGAR_v8 (annual maps varying interannually) and VISIT. The VISIT model simulated fluxes vary monthly and interannually, driven by reanalysed climate fields and fertiliser-use information from the global N_2_O Model Intercomparison Project, Phase 2 (NMIP2; Tian et al. 2018, 2024). The VISIT natural soil emissions contain effects of climate variability and agricultural soil emissions are dominantly controlled by nitrogen fertilisers, manure production and atmospheric deposition of reactive-nitrogen (N_R_) on land (Ito et al. 2018).

**Table 1:** List of emission combinations used in running the forward and inverse model cases. Combinations are prepared using industrial, natural soil, agricultural soil and sea-air N_2_O fluxes (column 2 through 5, respectively). Global total emissions for each of the distinct sectors are given in parenthesis as average values for the decade of 2000s and 2010s, respectively, for the interannually varying emission sectors (else only one long-term mean is given; units: TgN yr^− 1^). Values in the parenthesis are not repeated for each row, if data source is identical, and only one value is listed for the emissions without interannual variability

Abbreviated name of model case	Anthropogenic industrial emissions	Natural soil emissions	Agriculture soil/fertiliser-use emissions	Ocean/sea-air exchange
1. agey32	EDGAR_v8 (1.8, 2.0)	GEIA (7.5)	EDGAR_v8 (3.9, 4.4)	Yang_32 (3.2)
2. avey32	EDGAR_v8	VISIT(7.4, 7.5)	EDGAR_v8	Yang_32
3. agvy32	EDGAR_v8	GEIA	VISIT (3.4, 3.8)	Yang_32
4. avvy32	EDGAR_v8	VISIT	VISIT	Yang_32
5. avvy36	EDGAR_v8	VISIT	VISIT	Yang_36 (3.6)
6. avvy42	EDGAR_v8	VISIT	VISIT	Yang_42 (4.2)

## Results and discussion

**Fig. 2:**
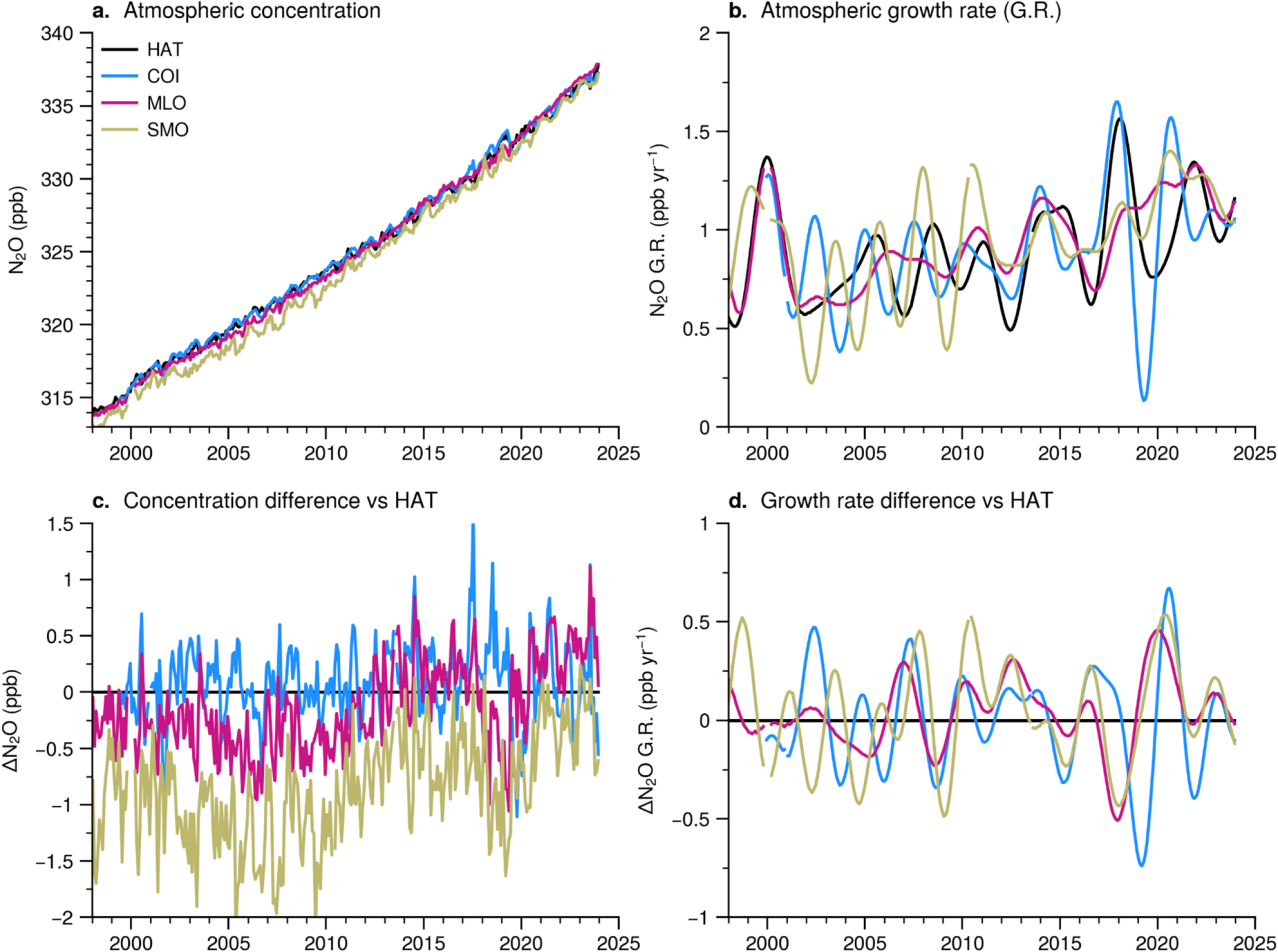
The NOAA and NIES observed N_2_O time series (**a**), atmospheric growth rate, G.R. (**b**), inter-site concentration gradients, ∆N_2_O, with respect to HAT (**c**), and differences in growth rates (∆N_2_O G.R.) with respect to HAT (**d**) are shown for selected sites during 1998–2024. The site locations can be seen in Fig. 1, at #41 (MLO; 19.53^o^N,155.58^o^W), #44 (SMO; 14.25^o^S,170.57^o^W), #48 (COI; 43.15^o^N,145.50^o^E) and #49 (HAT; 24.05^o^N,123.80^o^E)

The effect of regional emission signals is variedly captured by concentrations at different sites on the globe, producing dynamic spatial gradients. We present one such development in concentration gradient change in the East Asia and Pacific Ocean regions (with an aim to elucidate the emission processes involved using numerical models). Figure 2a shows the time series of N_2_O concentration, spreading over the downwind of continental East Asia (HAT: Hateruma and COI: Cape Ochi-Ishi, Japan) and remote islands in central tropical Pacific Ocean (SMO: American Samoa Observatory and MLO; Mauna Loa Observatory, USA). The growth rate time series for the period 2000 to 2023 suggest an acceleration of the growth rate in recent times, post 2017, without any sustained differences between sites (Fig. 2b, d). One of the aims of this study is to understand the causes of large systematic changes in inter-site concentration differences/gradients (∆N_2_O), greatest between SMO - HAT, and smallest between COI - HAT and intermediate between MLO - HAT (Fig. 2c). Detection of inter-site gradients and annualised growth rates, typically below 1.5 ppb, demands extremely high precision measurements and traceability of concentrations to the calibration scale, which has been the target of the research community. Despite all the quality controls, there is some doubt about the drastic changes in NIES measurements from COI and HAT in 2019, showing a sudden drop in concentration. However, this will not affect overall conclusions of this work (Fig. S1), but is highlighted as one of the important issues that the measurement community is striving to achieve for each of the measurement points.

There is no general consensus on what should be the acceptable difference in measurement scales between the institutions and how dense a measurement network is essential for regional flux inversion. To explore this, we performed 4 additional inversions by selecting 3 sets of the measurement networks, by excluding overlapping NOAA and AGAGE sites and by excluding 2 NIES sites, and by adopting a time-dependent scale adjustment for the AGAGE sites (Fig. S1). Since the NOAA cooperative measurement network covers most parts of the globe, the inversion estimated N_2_O emissions are not significantly different for the 15 land regions. The emission anomaly showed greater differences during 2012–2023 for the inversions with or without NIES sites at HAT and COI (Fig. S1i), as these sites on the western edge of Japan capture the outflow of mainland China emissions, critical for capturing the time evolution of continental fluxes (Tohjima et al. 2020). Note also that the mean of estimated N_2_O emissions is fairly similar for the inversion cases with and without the NIES sites, 1.90 and 1.88 TgN yr^− 1^, respectively. However, a much greater impact of up to 0.4 TgN yr^− 1^ lower N_2_O emissions is found for the East Asia region when calibration scale adjustment was not applied (Fig. S1; case: agey32_noScl). The time-dependent scale adjustment vs. fixed scale adjustment to AGAGE sites also did not produce significant difference in inversion fluxes at regional (Fig. S1) or at semi-hemispheric level (not shown). These results only reiterate the usefulness of measurement on accurate scales with intercomparability but cannot prescribe any level of tolerance.

Since N_2_O is a chemically inert species in the troposphere, the inter-site gradients in concentrations are likely to arise from the regional changes in surface fluxes, which are estimated by inverse modelling and discussed in the rest of the article. The effect of changing transport is accounted for by the transport model simulations using realistic meteorology by nudging MIROC4-ACTM meteorology to JRA-55. The N_2_O growth rates are known to vary closely with El Niño Southern Oscillation (ENSO) at decadal time scales due to natural processes such as changes in soil moisture, and atmospheric transport (Fig. 2b; Ishijima et al. 2009; Nevison et al. 2007; Saikawa et al. 2014). An acceleration in the growth to ~ 1.0 ppb yr^− 1^ has been seen during 2014–2018, compared to 0.7 ppb yr^− 1^ in 2001–2005, caused by anthropogenic activities (Thompson et al. 2019), which is further intensified to 1.15 ppb yr^− 1^ in 2019–2023. The difference in growth rates with respect to HAT only show interannual variability, while the mean growth rate differences are less than 0.04 ppb yr^− 1^ during 1998–2023 (Fig. 4d). This uniformity in long-term mean N_2_O growth rate is a result of no apparent loss on the Earth’s surface or in the troposphere, and the interannual variability in growth rate differences arise from timing of regional sources variability and their transport to sites at a range of distances.

**Fig. 3:**
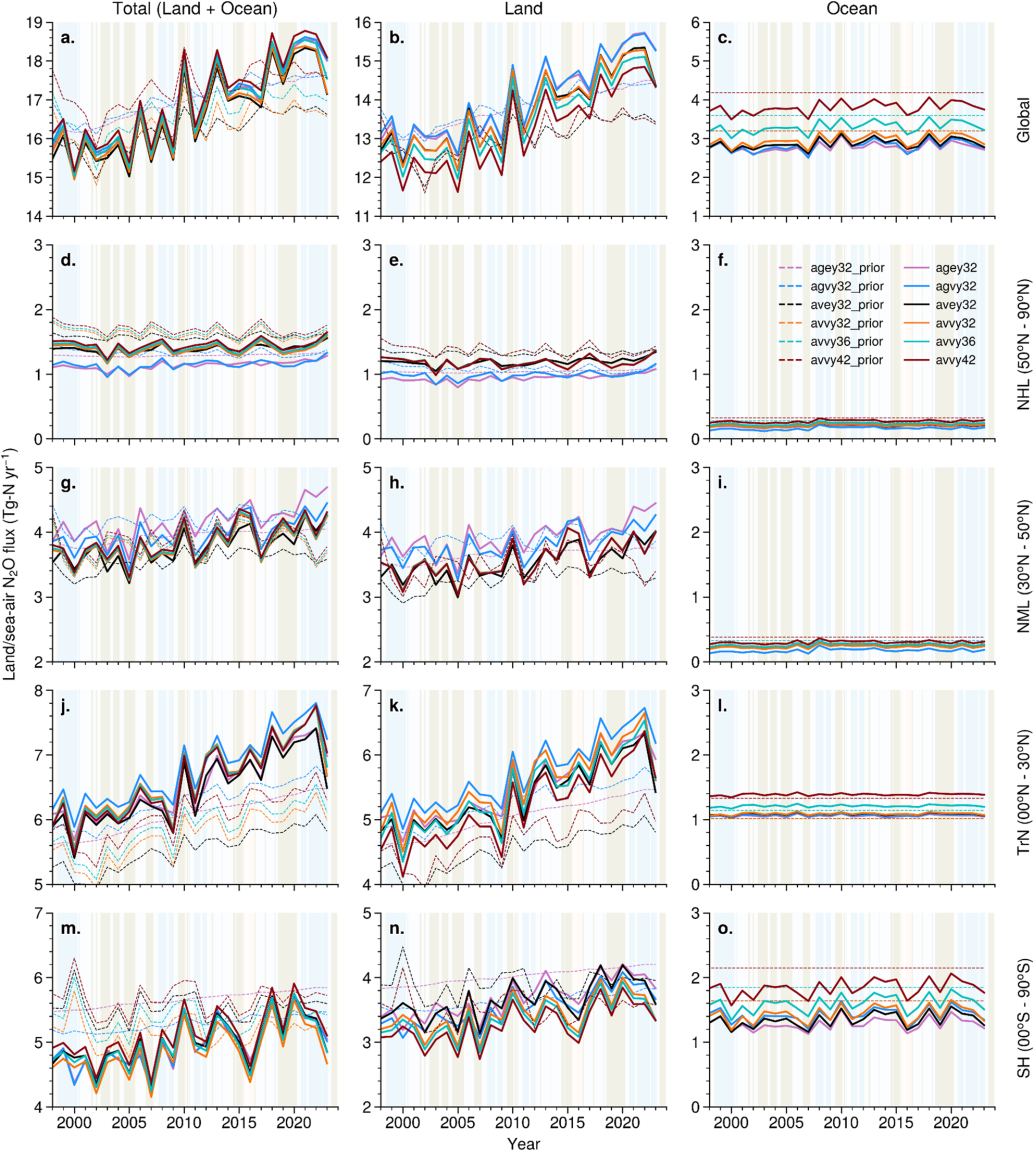
Global total and semi-hemispheric N_2_O flux time series of land and ocean (first column), land only (second column), and ocean only (third column) are shown at annual intervals for the period 1998–2023. Dotted lines represent prior mean flux estimates, while solid lines represent corresponding inversion-estimated fluxes. The land fluxes include both the anthropogenic (industrial + agriculture soil) and natural (soil) components. Note variable y-axis limits but a common y-axis range of 3 TgN yr^− 1^ for the semi-hemispheric fluxes are used for depicting relative strength of the interannual variabilities (panels d-o). The light blue and red shades denote La Niña and El Niño periods of ENSO, respectively

Figure 3 shows the time series of global and regional N_2_O emissions using atmospheric data from 49 sites in the inversions. Long-term mean emissions for the global land are somewhat consistent for the land regions in the prior and predicted cases (Fig. 3b), while the disagreements between the prior and predicted cases are clear for the latitude bands. Most prominently, the prior emissions are systematically underestimated for the NH tropics (TrN; Fig. 3k) lands, while the prior emissions are overestimated for the Southern Hemisphere (SH; Fig. 3n) land for the period earlier than 2010. Similar to the results obtained in Patra et al. (2022), our inversion system suggests that the long-term mean oceanic emissions should be lower than 3.2 TgN yr^− 1^ (3 lowest cases of ocean a priori emission) and inversions estimated the global total ocean emission to be 2.9 ± 0.15 TgN yr^− 1^ (Fig. 3c). The long-term variabilities are driven by industrial and agricultural soil emissions, whereas the interannual variabilities are driven by natural soil emissions (Fig. S2).

**Fig. 4:**
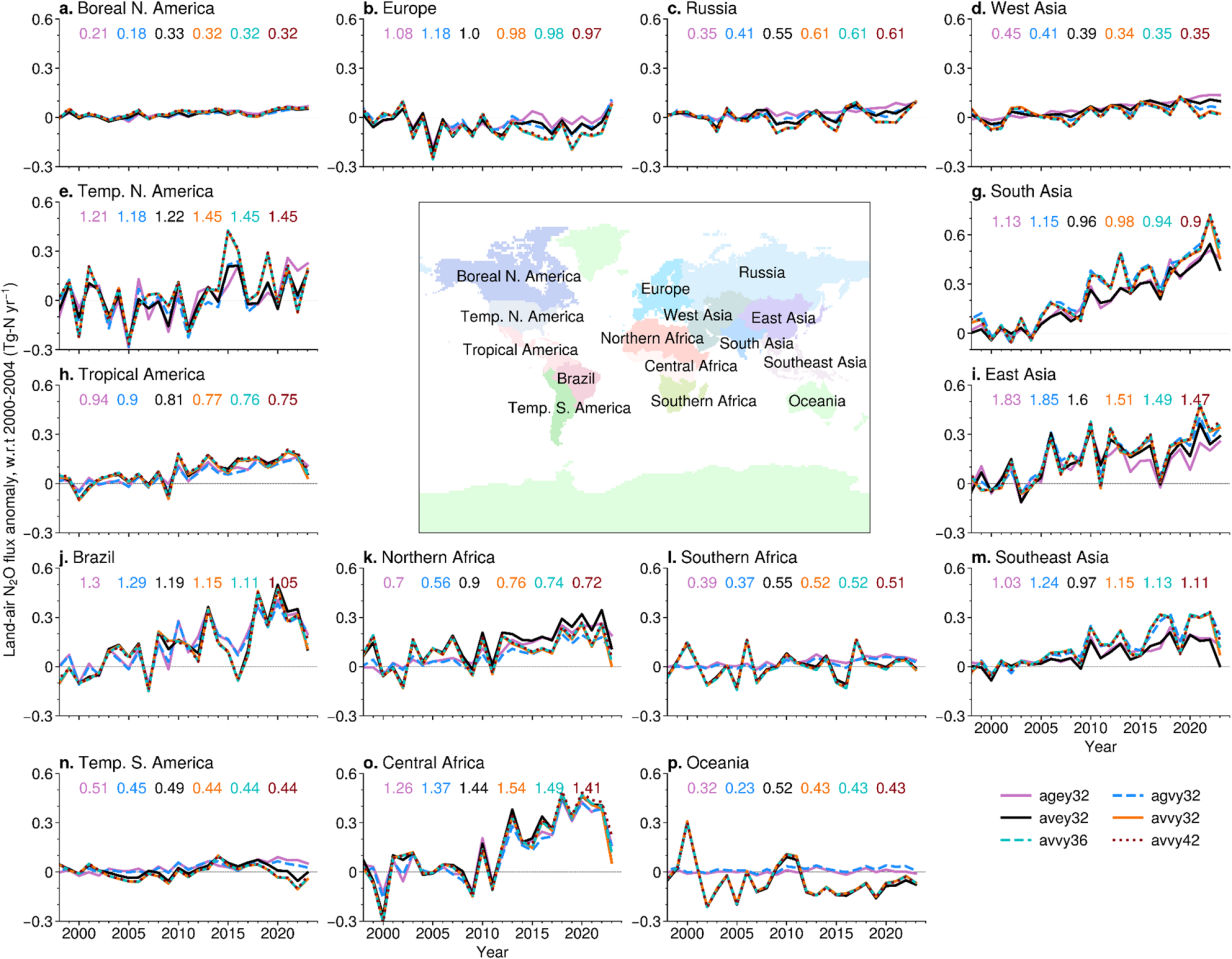
Monthly time series of regional N_2_O flux anomalies for the ensemble of inversions over 1998–2023 period, relative to the 2000–2004 mean. The number shown in each panel is the average for 2000–2004 for each individual inversion case. Sectoral emission anomalies for the land regions are shown in Supplementary Fig. S2. The flux anomaly time series of 11 ocean regions are shown in Supplementary Fig. S3

The control of ENSO driven climate anomalies on inversion estimated N_2_O flux variability is apparent for the global land (Fig. 3b), which generally show a peak following La Niña and trough following an El Niño (Thompson et al. 2014; Patra et al. 2022). The interannual flux variabilities will not be discussed in greater detail in this article and focus is given to emission change rates during different time periods and regions. However, the interannual variabilities in regional land and ocean fluxes are seen in Fig. 4 and Fig. S3, respectively, and a statistical analysis of lead-lag correlation on N_2_O flux anomalies at 3-monthly mean intervals are given in Table S2. It is found that the TrN and SH land regions are the main drivers of the global N_2_O emission anomaly and ENSO index variability (Table 2). Although the ocean regions also show statistically significant correlations (Table S2), the magnitude of variability in N_2_O fluxes are small compared to those for the land. This is under the assumptions of the inverse model setup, e.g., the a priori flux uncertainties are set at 50% of the net emissions for all 54 land and 30 ocean regions, and the 49 sites in the observational network (ref. Sect. 2).

We find the global total emission increase occurred strongly during 2005–2018 and then stabilised at the highest level in the recent years (2018–2023). The major interannual variabilities and trends on global N_2_O emissions are discussed in details later in the article. Compared to all the prior flux cases (50 ± 10 GgN yr^− 1^ per year) the global land emissions increased at a much faster rate of 106 ± 5 GgN yr^− 1^ per year for the inversion fluxes during the period 1998–2002 (first 5 years) and 2019–2023 (last 5 years) of analysis. The uncertainty of emission increase rates are calculated based on 6 inversion cases, and the increase rate represents the difference between mean fluxes in 1998–2002 and 2019–2023, divided by the time interval. Inversion cases only marginally, but persistently, increased N_2_O emissions from global oceans (7 ± 2 GgN yr^− 1^ per year), from no prior flux variability, from 1998 to 2002 to 2019–2023. This small increase in oceanic emission could arise from the deposition of N_R_ on ocean surfaces (e.g., Duce et al. 2008), which has remained fairly stable globally since the 1990s (Tian et al. 2024; their Fig. B3).

The origin of land flux variations and trends are found to occur from the northern hemisphere (NH) mid-high latitudes (NML; Fig. 3h), TrN (Fig. 3k) and SH (Fig. 3n) lands. However, the rate of increase in N_2_O emissions varies with time from these 3 latitude bands with distinct phases of flux variability, which can explain the inter-site concentration gradient between HAT and SMO, as discussed earlier (Fig. 2c). As expected, the spread between the inversion estimated fluxes decreased for all the latitude bands, compared to the spread in 6 a priori emission cases. The SMO-HAT ∆N_2_O decreased during 1998–2007 due to faster increase in emissions from the NML and TrN (5.7 ± 8.7 and 34 ± 3.3 GgN yr^− 1^ per year, respectively; Fig. 3e, k), compared to that from the SH land (1.6 ± 9 GgN yr^− 1^ per year; Fig. 3n). This SMO-HAT ∆N_2_O trend was reversed in the period 2007 through 2023 as the emission increase in the SH land gained faster pace (28 ± 7 GgN yr^− 1^ per year), comparable to that for the NML (33 ± 12 GgN yr^− 1^ per year) and TrN (74 ± 21 GgN yr^− 1^ per year).

**Table 2:** Statistics of agreement between VISIT simulated natural + agricultural soil N_2_O fluxes (avvy32 prior cases) with the inversion estimated flux cases (a**y32) for semi-hemispheric land divisions (ref. Fig. 3), indicating the capacity of the inverse model to retrieve N_2_O flux variability and consistency of VISIT simulated fluxes with observed N_2_O in the atmosphere. The N_2_O emission anomaly and ENSO index correlations calculated using 3-monthly mean time intervals data (right-most column), with detailed in Table S2

Semi-hemispheric land divisions	Spearman’s rank Correlation (*r*) / *p*-value of inversion cases and VISIT prior case (avvy32)				Change in emissions, (2019–2023) – (1998–2002)	*r* for mean of all 6 cases with ENSO
	agey32	agvy32	avey32	avvy32		
Global total	0.70/ 0.0001	0.72/ 0.0001	0.72/ 0.0001	0.77/ 0.0001	2.32	− 0.26
NH high latitudes (NHL)	− 0.04/ 0.84	0.45/ 0.02	0.38/ 0.051	0.78/ 0.000	− 0.02	− 0.20
NH mid-high latitudes (NML)	0.33/ 0.08	0.41/ 0.03	0.37/ 0.06	0.50/ 0.001	0.39	0.07
NH tropics (TrN)	0.84/ 0.0001	0.88/ 0.0001	0.84/ 0.0001	0.89/ 0.0001	1.35	− 0.28
SH total (SH)	− 0.09/ 0.66	0.09/ 0.63	0.49/ 0.01	0.51/ 0.01	0.59	− 0.39

Figure 4 shows that the regional land flux anomalies exhibit large variations as well as increasing trends for the regions where agriculture is intensive, suggesting that the global use of nitrogen fertiliser and manure has grown in the past two decades. The emission anomalies for industrial anthropogenic, agricultural soils and natural soils along with the inversion ensemble means are shown in Fig. S2. The interannual variability for the tropical regions is controlled by the dominant climate variability driven by ENSO, as discussed in Patra et al. (2022, and references therein). It is noted that the VISIT model simulated (bottom-up) prior flux interannual variabilities (cases: avvy32) are generally well retrieved by the inversions (top-down) for a priori that used no interannual variability (case: agey32) at the semi-hemispheric divisions (Table 2). Such top-down and bottom-up agreements are also apparent for most of the 15 land regions (Fig. 4), the level of which defines our ability to comprehensively understand N_2_O emission processes. The Oceania region fluxes using VISIT natural soil are not supported by inversions (cases: av* vs. cases: ag*; Fig. 4p), which is likely due to the fact that the Australian measurement network does not constrain the land fluxes (Haverd et al. 2013). Similar is the case with Southern Africa, where the interannual variability in VISIT natural soil is not estimated solely by inversion (Fig. 4l). It is to be acknowledged here that high quality data are available at more sites in the post-2010 period, but are excluded in the inversions as this analysis aimed to cover results since the late 1990s.

The regions with net emissions exceeding 1 TgN yr^− 1^ during 2000–2004 are Europe (Fig. 4b), Temperate North America (Fig. 4e), South Asia (Fig. 4g), East Asia (Fig. 4i), Brazil (Fig. 4j), Southeast Asia (Fig. 4m) and Central Africa (Fig. 4o). Among these, only Europe showed a stable or a slightly declining trend in emissions during our analysis period (1998–2023), suggesting the use of nitrogen fertilisers and manure in the major agricultural active nations continues to grow. The other regions showing fast increasing emissions are Tropical America (Fig. 4h) and Northern Africa (Fig. 4k), with total emissions touching 1 TgN yr^− 1^ in the 2020s. Figure S4 shows the net amount of nitrogen fertilisers used in each region along with the ensemble mean net N_2_O emissions by inversions. The ratios of regional total (natural+anthropogenic) N_2_O emissions to nitrogen fertiliser use are found to be about 0.1 for the agriculture intensive regions in the recent years (e.g., the 2020s), while the regions with a large fraction of uninhabited lands (greater fraction of natural soil) show the emission ratios exceeding 0.2 (ref. Fig. S5). Control of the fraction of nitrogen compounds released as N_2_O from the agricultural soils is one of the options to reduce N_2_O emissions (Shen et al. 2021; Mosier et al. 1998; Lawrence et al. 2021).

The regional N_2_O emission intensity (defined by the ratio of total emissions to anthropogenic nitrogen input) is found to be stable over Europe, Temperate North America, Tropical America and West/South/East Asia, while the other 9 regions showed a reduction in the ratio during 1999–2023. The atmospheric deposition of N_R_ has experienced drastic changes during the period of analysis, e.g., over the United States (Zhu et al. 2025a, b), but likely to be poorly accounted in driving the global biogeochemical models (Tian et al. 2024). However, it could be argued that this drastic change in N deposition is largely driven by agricultural activities (Galloway et al. 2003; Chen et al. 2020), meaning that the temporal change in N_2_O emissions is directly or indirectly attributable to the use of N fertilizers. The use of nitrogen fertilisers applied to cropland and pasture, and manure additions to cropland, pasture and rangeland have continued to rise (ref. Tian et al. 2024). In the VISIT model, about 50% of the emission increase since the early 2000s is caused by nitrogen fertiliser use, and a major part of the remaining half is caused by manure application and atmospheric N_R_ deposition in Asia (Ito et al. 2018). A clear separation of sector-wise emissions is not possible by our inverse model due to coarse forward and inversion model resolutions, and thus the determination of regional emission factor for nitrogen fertilisers would be overestimated if based on total regional inversion fluxes.

**Fig. 5:**
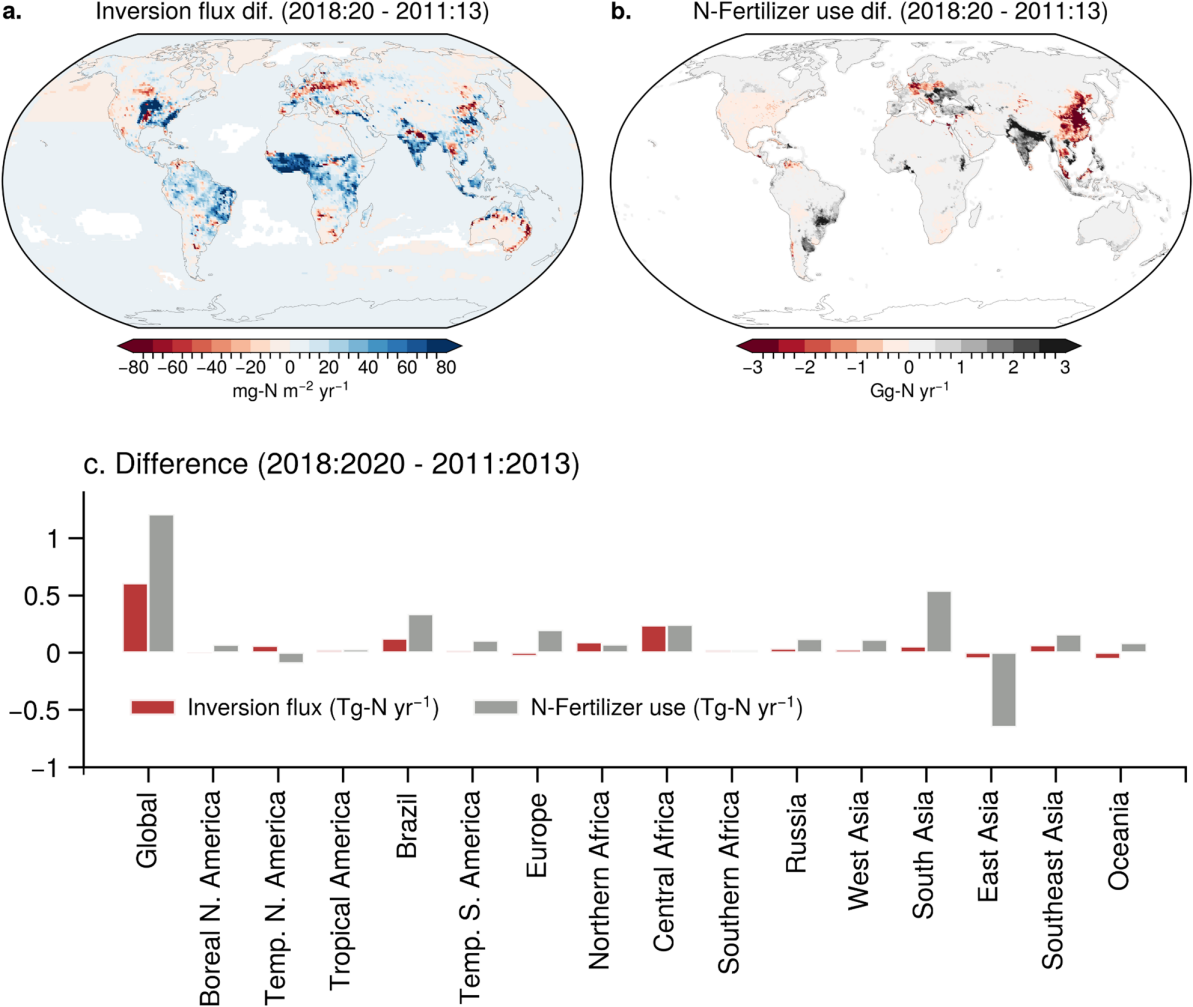
The spatial maps illustrate the change in mean inversion fluxes, including all sectors (**a**) and nitrogen fertiliser applied to cropland and pasture (**b**) between the periods 2018–2020 and 2011–2013 (differences over 7 year). The bar charts (**c**) display the regional changes in fluxes, as depicted in the spatial maps above. Note that this analysis is restricted until 2020 because of the availability of N-fertilizer input data. Results for the 2016–2020 vs. 2000–2004 are shown in Fig. S6. The correlations between the differences in inverse model fluxes and nitrogen fertliser use are shown in Fig. S7

Figures 5 and Fig. S6 show how the regional N_2_O emissions from all source sectors and nitrogen fertiliser use have changed over time, the most recent decade and over the period of the analysis, respectively. In the period from 2000 to 2004 to 2016–2020 all regions showed increased fertiliser use and thus increased N_2_O emissions, except for some parts of Europe, while the maps of changes in the period from 2011 to 2013 to 2018–2020 show large regional variations. Most prominently, the nitrogen fertiliser use has decreased over China, Europe and Temperate North America (Fig. 5b). As a result, some regions of China are exhibiting decreases in N_2_O emissions (Fig. 5a), with the East Asia region as a whole showing a decrease in emissions (Fig. 5c). In contrast, most parts of South Asia and temperate regions of South America show a continued increase in fertiliser and manure use and N_2_O emissions in the most recent decade. Although no large increase in fertiliser use is seen over most parts of Africa, our inversion suggested large increases in emission, in particular over the tropical regions. Notably these areas of tropics are also the regions of large fraction of manure application to cropland (Tian et al. 2018). More careful separation of the natural and agricultural soil emissions must be made before we can arrive at more concrete conclusions on NUE of nitrogen-fertilisers using atmospheric inversions, including that of the cropland expansion/reduction.

## Conclusions

We have performed inverse modelling of atmospheric N_2_O for the period of 1998 to 2023 using measurements made by NOAA, AGAGE, CSIRO, NIES and JMA operated sites at 40 distinct locations worldwide (49 sites in total). The inverse model uses the emission inventories from EDGAR_v8, VISIT land ecosystem model, GEIA natural soil emissions, and oceanic exchange maps. Global mean N_2_O has increased at the rates of 0.8 and 1.1 ppb yr^− 1^ in the first (1998–2007) and final (2014–2023) decades of our analysis, respectively, with corresponding global total emissions of 15.7 ± 0.5 and 17.7 ± 0.6 TgN yr^− 1^. The increase rate of total land emissions by inversions (106 ± 5 GgN yr^− 2^) is found to be almost twice as large as those in the case of prior emissions (50 ± 10 GgN yr^− 2^), suggesting a gap in process level understanding of N_2_O emissions that is required for projecting future emission scenarios. With the current prior and observational network, the observations can be reproduced without requiring large additional trends in the ocean flux.

The observations since 2010 suggest that N_2_O concentration differences have lessened between HAT (24^o^N, located in the emission outflow region of continental East Asia) and two tropical sites in the central Pacific Ocean (SMO; 14^o^S and MLO; 19^o^N). Correspondingly, the inversion modelling estimated faster increase in the NH tropical and SH lands, compared to the NH mid and high latitude lands. At the regional scales, 13 out of 15 land regions show moderate to fast increase in emissions, suggesting only the Europe and Oceania regional emissions have stayed stable in the period of our analysis. We show the increase in regional N_2_O emissions are closely linked to the agricultural activities by the extensive use of nitrogen fertilisers.

Based on the VISIT ecosystem model and NMIP simulations, FAO statistics of fertiliser use, and inversion estimated regional emission trends suggest that the ratio of N_2_O emission to nitrogen fertiliser use have changed over time. The N_2_O emission intensity (the ratio) is found to be stable over Europe, Temperate North America, Tropical America and West/South/East Asia, while the other 9 regions showed a reduction in the ratio. In the recent decade, nitrogen fertiliser use and N_2_O emissions both have stabilised or slightly decreased in East Asia, mainly due to the changes over China. Emissions of N_2_O from South Asia, Southeast Asia, Brazil, and Central and Northern Africa regions showed the fastest increase in the past two decades, where the use of both nitrogen fertlisers and manure continued to rise.

## Supplementary Information

Below is the link to the electronic supplementary material.

Supplementary Material 1

## Data Availability

The N 2 O inversion dataset generated and analysed in the current study are available in the Zenodo repository [https://doi.org/10.5281/zenodo.14533031]. VISIT ecosystem model simulation of N 2 O flux are available at : ZenodoEDGAR_v7 data are available at: [https://edgar.jrc.ec.europa.eu/dataset_ghg80](https:/edgar.jrc.ec.europa.eu/dataset_ghg80)The processed datasets are also available from the corresponding author on reasonable request.
