# Supplementary material for: Recent increase in N2O growth rate (2013–2023) mainly due to increase of nitrogen-fertiliser and manure use in the Northern Tropics and Southern Landmass
Source: Geosci Lett. 2026 Apr 28;13(1):27. doi: 10.1186/s40562-026-00476-z (PMC13124763; doi:10.1186/s40562-026-00476-z)
Supplement: Supplementary file 1 — Supplementary Material 1 [file 40562_2026_476_MOESM1_ESM.pdf]

Supplementary materials for:

Recent increase in N_2O growth rate (2013-2023) mainly due to increases of nitrogen-fertiliser and manure use in the northern tropics and southern landmass

Prabir K. Patra[1,2,3,], Yasunori Tohjima[4], Akihiko Ito[5], Naveen Chandra[1], Motoki Sasakawa[4], Xin Lan[6,7], Bradley D. Hall[6], Paul B. Krummel[8], Ray F. Weiss[9], Christina M. Harth[9], Shinya Takatsuji[10], Daisuke Goto[11], Kumiko Takata[1], Luke M. Western[12], Ronald G. Prinn[12]*

1. Research Institute for Global Change, JAMSTEC, Yokohama, 236-0001, Japan

2. Seto Inland Sea Carbon Neutral Research Center, Hiroshima University, Hiroshima 739-8529, Japan

3. Research Institute for Humanity and Nature (RIHN), Kyoto, 603-8047, Japan

4. Earth System Division, National Institute for Environmental Studies (NIES), Tsukuba 305-8506, Japan

5. Graduate School of Agricultural and Life Sciences, The University of Tokyo, Tokyo 113-8657, Japan

6. Global Monitoring Laboratory, National Oceanic & Atmospheric Administration (NOAA), Boulder, CO 80305, USA

7. Cooperative Institute for Research in Environmental Sciences, University of Colorado Boulder, CO 80309, USA

8. CSIRO Environment, Aspendale, Victoria 3195, Australia

9. Scripps Institution of Oceanography, University of California, San Diego, CA 92093, USA

10. Atmospheric Environment and Ocean Division, Atmosphere and Ocean Department, Japan Meteorological Agency (JMA), Tokyo 105-8431, Japan

11. National Institute of Polar Research, Tachikawa, Tokyo, 190-8518, Japan

12. Massachusetts Institute of Technology, Cambridge, MA 02139, USA

* Corresponding author e-mail : prabir@jamstec.go.jp

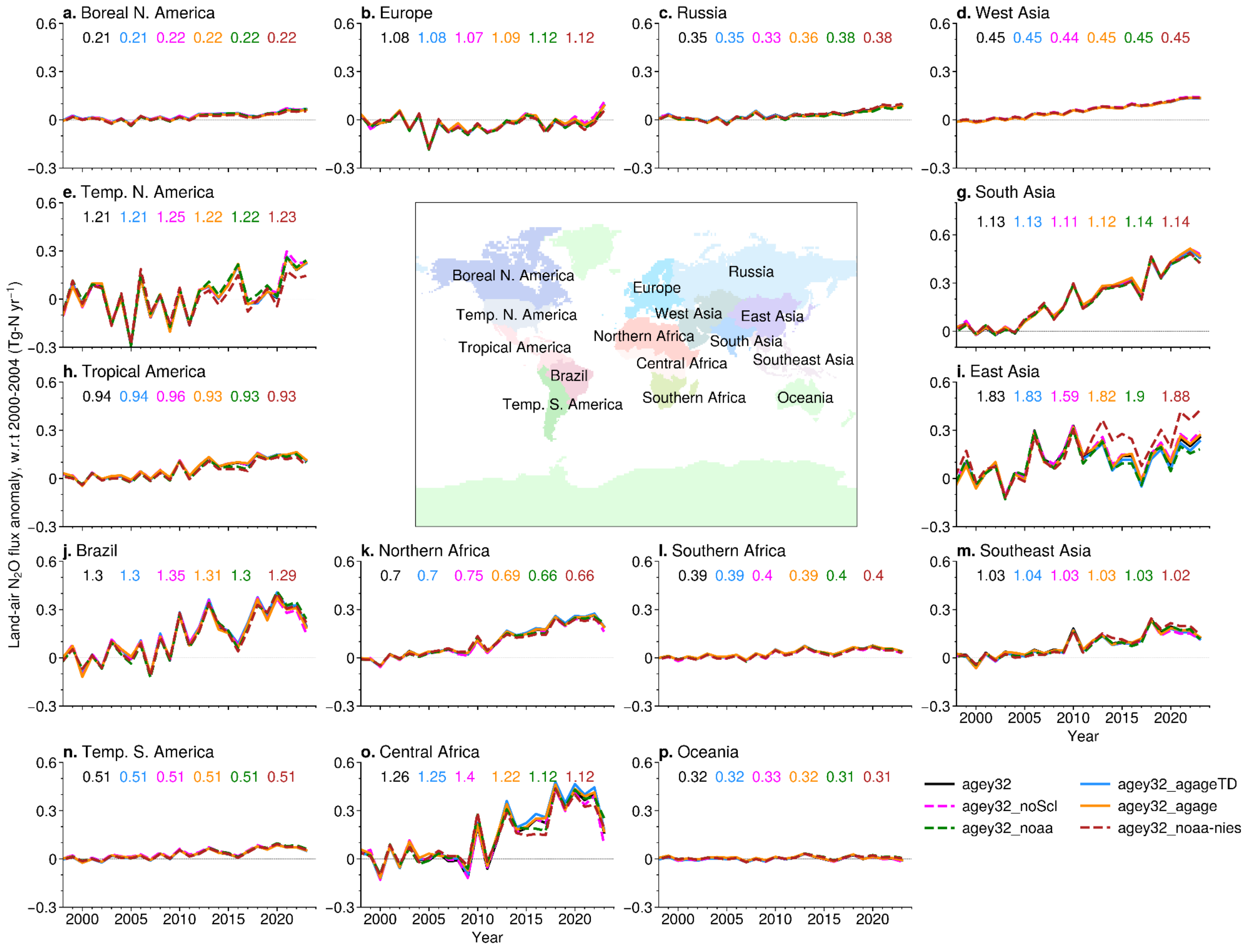

Figure S1: Same as Figure 4, but inversions show the effect of NOAA vs AGAGE vs NIES network differences, as well as, the impacts of AGAGE scale selection. The **control case (agey32)** here used all 49 sites data in inversion using "fixed" adjustments for the inter-institutional scale differences, while the **case - agey32_agageTD** used the "time-dependent" scale correction to the AGAGE sites, i.e., NOAA/AGAGE = 4.3641x10^{-5} * Year + 0.91065 and **case - agey32_noScl** used no scale adjustments. The **case - agey32_agage** excluded 5 NOAA sites (n2o_alt_surface-flask_1, n2o_cgo_surface-flask_1, n2o_mhd_surface-flask_1, n2o_rpb_surface-flask_1, n2o_smo_surface-flask_1), **case - agey32_NOAA** excluded 10 sites of AGAGE and other institute (n2o_alt_surface-flask_16, n2o_brw_surface-insitu_2, n2o_cgo_surface-flask_16, n2o_cgo_surface-insitu_4, n2o_mhd_surface-insitu_4, n2o_mlo_surface-flask_16, n2o_rpb_surface-insitu_4, n2o_smo_surface-insitu_4, n2o_spo_surface-flask_16, n2o_thd_surface-insitu_4), and **case - agey32_NOAA-NIES** excluded 12 sites as in the case of agey32_NOAA + 2 NIES sites (COI_N2O and HAT_N2O). The first 3 cases dealt with measurement scales (49 sites) and final 3 cases show impacts of measurement site networks (fixed scale adjustment).

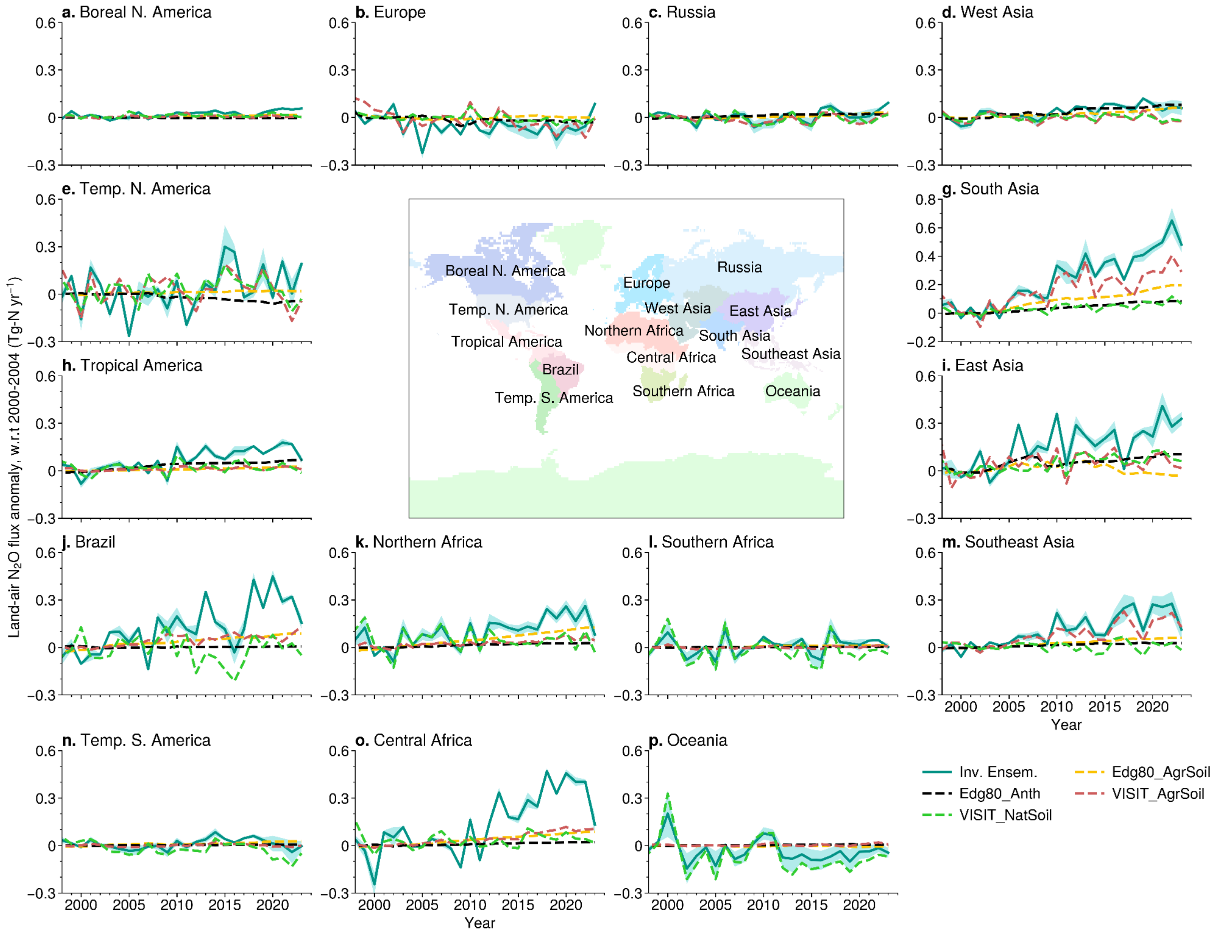

Figure S2: Ensemble mean and ±1σ spread (cyan line and shading) of N_2O flux anomalies of 6 inversion cases are shown, and with sectoral fluxes for 4 categories, namely, EDGARv8.0 agriculture soil (Edg80_AgrSoil), EDGARv8.0 anthropogenic/industrial (Edg80_Anth), and VISIT model simulated agriculture soil (VISIT_AgrSoil) and natural soil (VISIT_NatSoil). The GEIA natural soil emissions do not contain interannual variations.

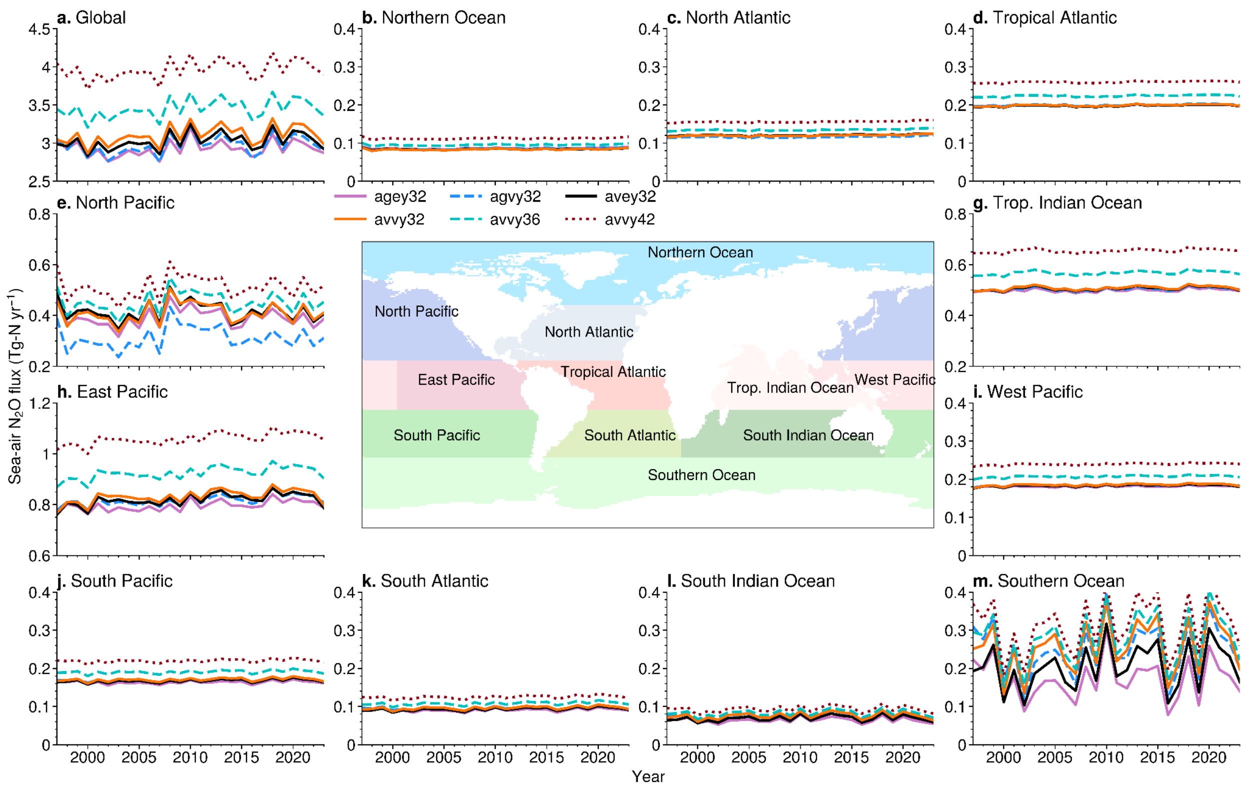

Figure S3: Same as Figure 3, but flux time series for the 11 ocean regions are shown.

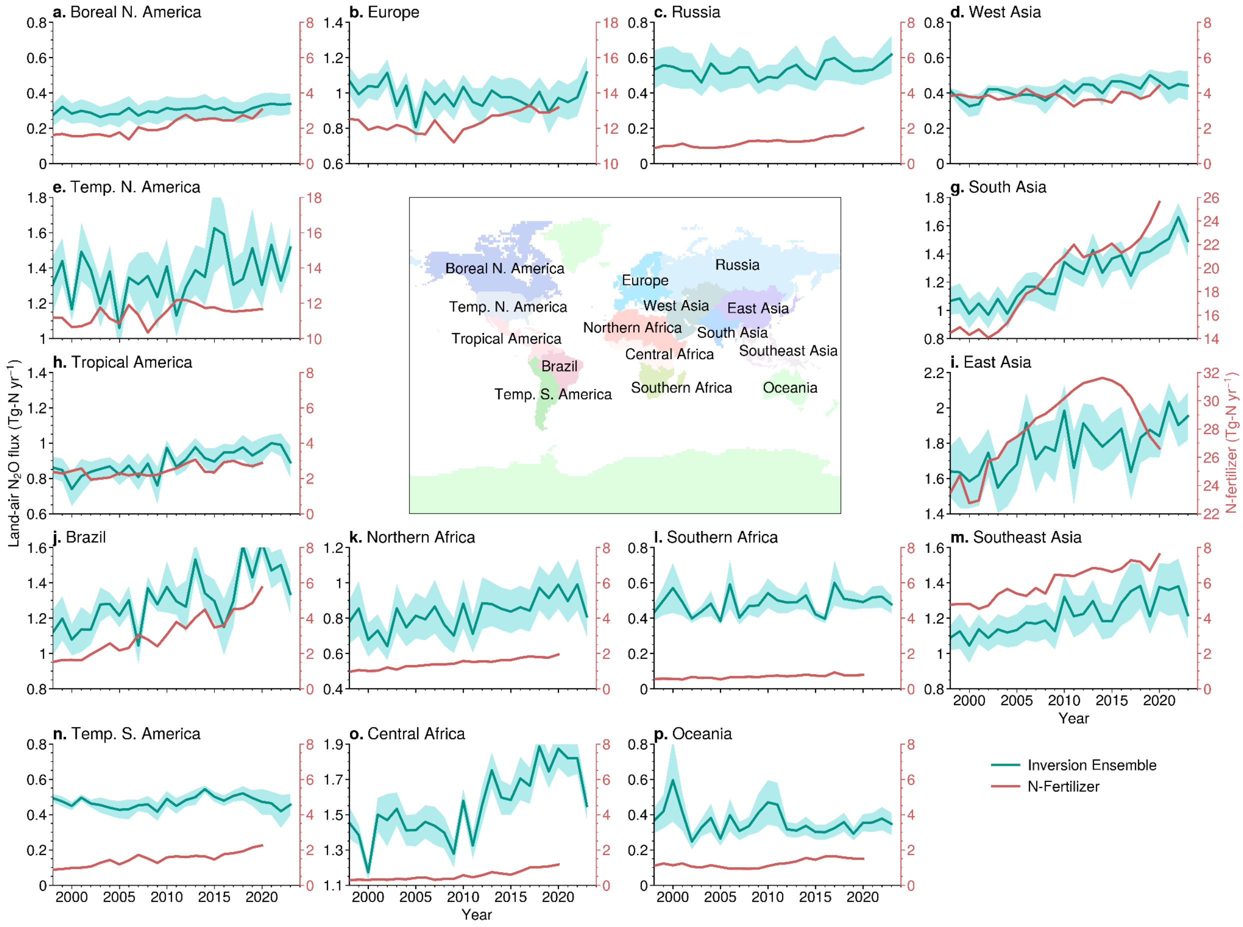

Figure S4: Annual mean time series of N_2O total emissions based on ensemble mean and ±1σ spread (cyan line and shading) of different inversion cases for the 15 regions depicted by central map. Nitrogen fertilizer used in the regions are also shown (N-fertilizer = crop_nh4 + crop_no3 + pasture_nh4 + pasture_no3 (red line). The anomalies in ensemble mean and spread between the inversions are shown in Fig. 5 and Fig. S4.

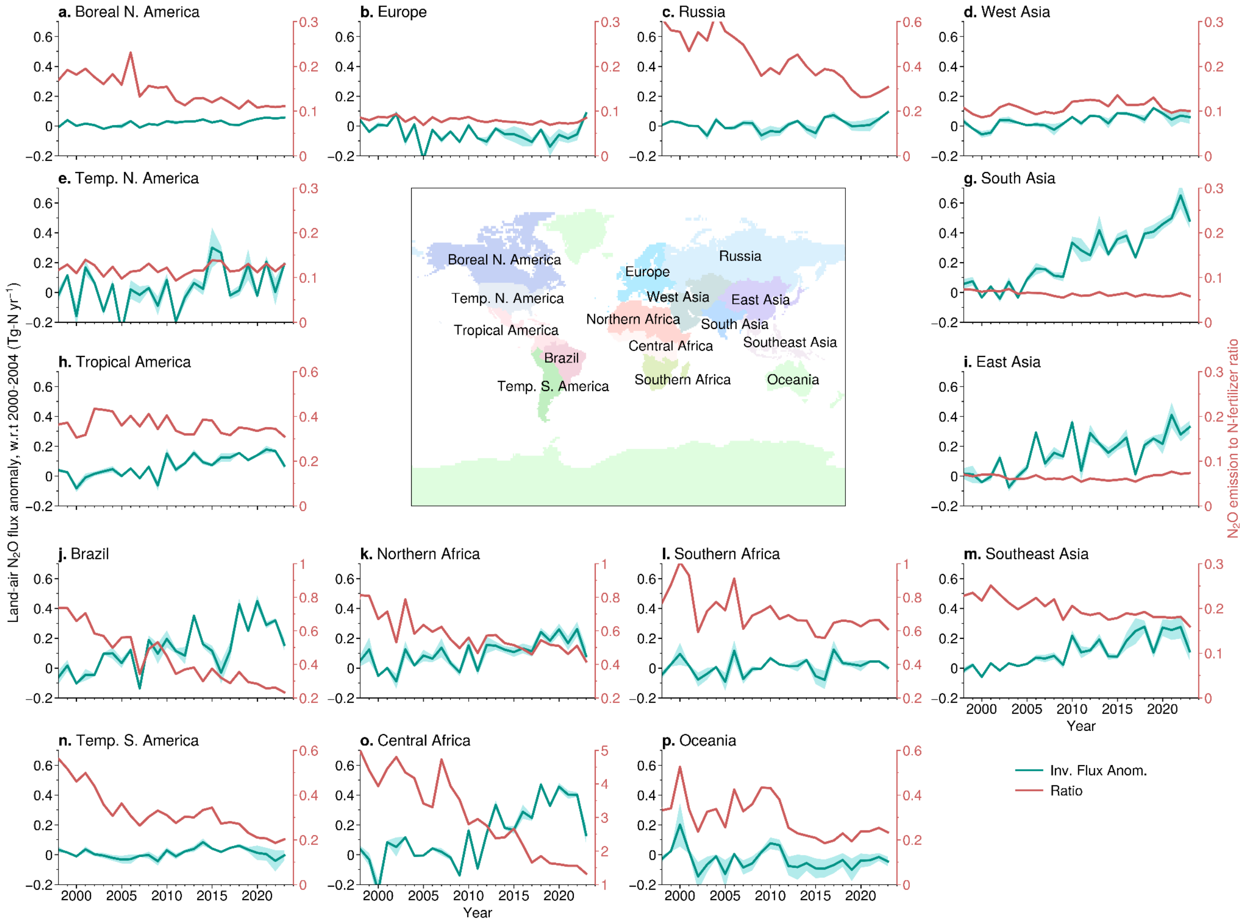

Figure S5: Same as Figure S2, but the annual mean anomalies in N_2O flux and ratio of N_2O flux to N-fertilizer use time series are shown. The flux anomalies are calculated relative to the 2000-2004 mean. The N-fertilizer use is assumed to remain constant at 2020 level for the later 3 years.

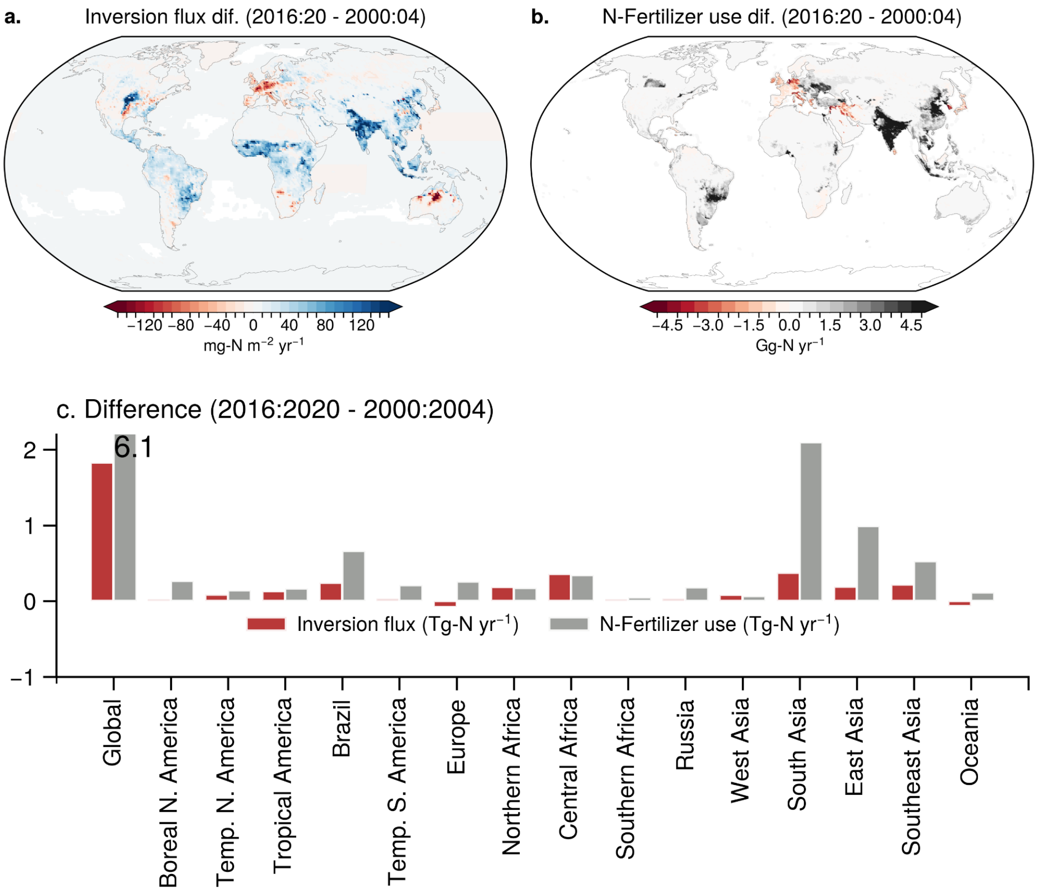

Figure S6: Same as Figure 7, but the N_2O flux and N-fertilizer use for the longer period of analysis are shown (2016-2020 and 2000-2004; differences over 16 years). Note that this analysis is restricted until 2020 because of the availability of N-fertilizer input data. Note that the increase in Global N-fertilizer use during the period this analysis is 6.1 as marked on the grey bar and a smaller y-axis range is chosen for clearly showing the regional differences.

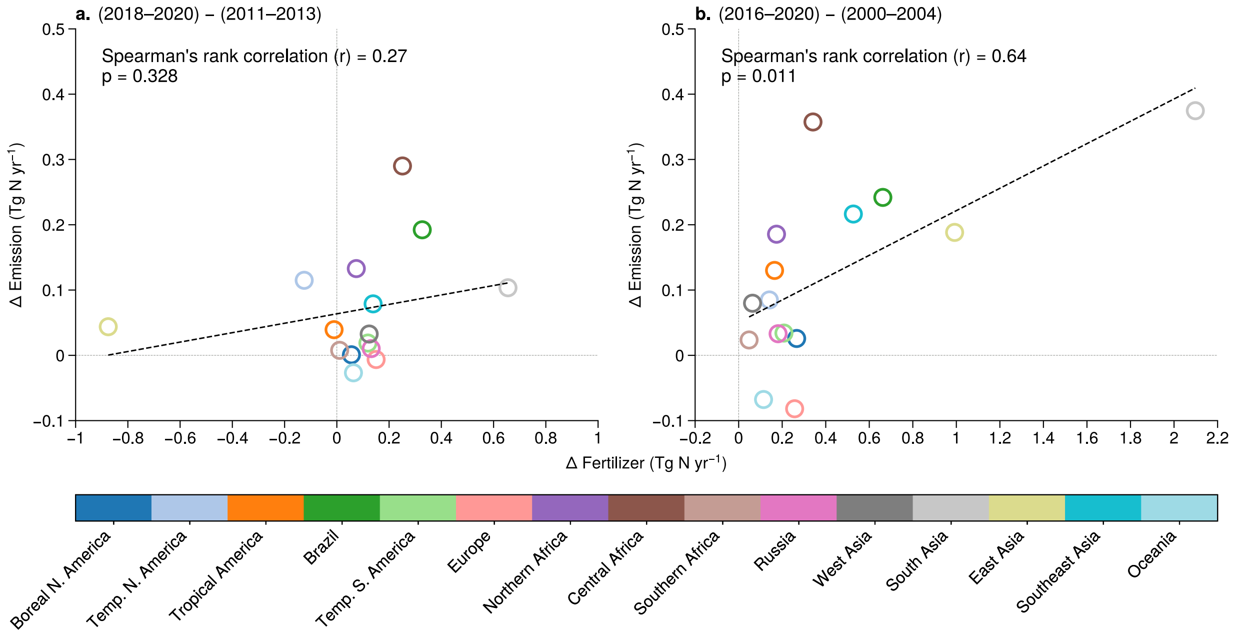

Figure S7: Scatter plots and correlation statistics of N2O emission and nitrogen fertiliser use change in the two periods of analysis, as in Fig. 5 (a) and Fig. S6 (b). Although a linear relationship does appear but the significance is low because of the complex nature of the N_2O emissions relating to nitrogen fertiliser use, e.g., soil type, crop types, land management etc. The map and bar plots (Fig. 5 and S6) are also essentially showing similar conclusions. A separation between agricultural land and natural soil must be done to better resolve the relationship but the current coarse resolution setups for forward and inverse models doesn’t allow the separation clearly (as mentioned in the manuscript).

Table S1. List of sites used in the N_2O inversion as available from NOAA/GML, WDCGG-AGAGE and NIES. Operating institute's name is given at the end of Site name (column 2).

Sl. No.	Site name and operating institution	Latitude	Longitude	Altitude
1	n2o_alt_surface-flask_1_NOAA	82.5	-62.5	190
2	n2o_asc_surface-flask_1_NOAA	-8.0	-14.4	90
3	n2o_ask_surface-flask_1_NOAA	23.3	5.6	2715
4	n2o_azr_surface-flask_1_NOAA	38.8	-27.4	24
5	n2o_bmw_surface-flask_1_NOAA	32.3	-64.9	51
6	n2o_brw_surface-flask_1_NOAA	71.3	-156.6	16
7	n2o_cba_surface-flask_1_NOAA	55.2	-162.7	57
8	n2o_cgo_surface-flask_1_NOAA	-40.7	144.7	164
9	n2o_crz_surface-flask_1_NOAA	-46.4	51.9	202
10	n2o_gmi_surface-flask_1_NOAA	13.4	144.7	5
11	n2o_hba_surface-flask_1_NOAA	-75.6	-26.2	35
12	n2o_hun_surface-flask_1_NOAA	47.0	16.7	344
13	n2o_ice_surface-flask_1_NOAA	63.4	-20.3	122
14	n2o_izo_surface-flask_1_NOAA	28.3	-16.5	2378
15	n2o_key_surface-flask_1_NOAA	25.7	-80.2	6
16	n2o_kum_surface-flask_1_NOAA	19.6	-154.9	5
17	n2o_mhd_surface-flask_1_NOAA	53.3	-9.9	26
18	n2o_mid_surface-flask_1_NOAA	28.2	-177.4	15
19	n2o_mlo_surface-flask_1_NOAA	19.5	-155.6	3402
20	n2o_nwr_surface-flask_1_NOAA	40.1	-105.6	3526
21	n2o_psa_surface-flask_1_NOAA	-64.9	-64.0	15
22	n2o_rpb_surface-flask_1_NOAA	13.2	-59.4	20
23	n2o_sey_surface-flask_1_NOAA	-4.7	55.5	7
24	n2o_shm_surface-flask_1_NOAA	52.7	174.1	28
25	n2o_smo_surface-flask_1_NOAA	-14.3	-170.6	60
26	n2o_spo_surface-flask_1_NOAA	-89.0	-24.8	2815
27	n2o_syo_surface-flask_1_NOAA	-69.0	39.6	19
28	n2o_tap_surface-flask_1_NOAA	36.7	126.1	21
29	n2o_ush_surface-flask_1_NOAA	-54.9	-68.3	32
30	n2o_uta_surface-flask_1_NOAA	39.9	-113.7	1332
31	n2o_uum_surface-flask_1_NOAA	44.5	111.1	1012
32	n2o_wis_surface-flask_1_NOAA	30.0	35.1	156
33	n2o_zep_surface-flask_1_NOAA	78.9	11.9	479
34	n2o_brw_surface-insitu_2_NOAA	71.3	-156.6	16
35	n2o_cgo_surface-insitu_4_AGAGE	-40.7	144.7	164
36	n2o_mhd_surface-insitu_4_AGAGE	53.3	-9.9	8
37	n2o_rpb_surface-insitu_4_AGAGE	13.2	-59.4	20
38	n2o_smo_surface-insitu_4_AGAGE	-14.2	-170.6	60
39	n2o_thd_surface-insitu_4_AGAGE	41.1	-124.2	2
40	n2o_alt_surface-flask_16_CSIRO	82.5	-62.3	210
41	n2o_cgo_surface-flask_16_CSIRO	-40.7	144.7	164
42	n2o_cya_surface-flask_16_CSIRO	-66.3	110.5	55
43	n2o_maa_surface-flask_16_CSIRO	-67.6	62.9	42
44	n2o_mlo_surface-flask_16_CSIRO	19.5	-155.6	3435
45	n2o_mqa_surface-flask_16_CSIRO	-54.5	158.9	13
46	n2o_spo_surface-flask_16_CSIRO	-89.0	-24.8	2847
47	n2o_ryo_surface-insitu_1_JMA	39.0	141.8	280
48	n2o_coi_surface-insitu_NIES	43.2	145.5	101
49	n2o_hat_surface-insitu_NIES	24.1	123.8	47

Table S2. Statistics of ENSO index and 3-monthly mean N_2O flux anomalies. A long-term mean seasonal cycle (1999-2023) is subtracted from the region-aggregated flux time series at monthly intervals. Then 3 monthly-mean time series of N_2O emissions and ENSO index are used in preparing the statistics. This table is presented to discuss how inversions using priors without interannual variations start to produce similar semi-hemispheric emission variabilities as those simulated by the VISIT model. Note that the VISIT model is run using reanalysed climate variables (Ito et al., 2018). There are several delays between the climate conditions or fertiliser use and N_2O emissions, and we cannot gain a good level of understanding through a simple multivariate analysis as compared to using the VISIT model results. Because the N_2O emissions following the application of fertilisers or manure or atmospheric N_R deposition has a time delay which varies from region to region depending on crop types and how the fertilisers are treated into the soil (this situation of N_2O flux variability is quite different from that of CO_2 where the climate variations are easily associated with regional flux variability; e.g., Patra et al., GBC, 2005; https://doi.org/10.1029/2004GB002258).

Regions	ENSO vs N_2O (lag (months), r, p)				Mean r
	agey32	agvy32	avey32	avvy32	
Global Land	-2, -0.15, 0.01	-2, -0.17, 0.00	-2, -0.19, 0.00	-2, -0.21, 0.00	-0.18
Land_NHL	2, 0.09, 0.12	-2, -0.17, 0.00	-2, -0.04, 0.54	-2, -0.21, 0.00	-0.08
Land_MHL	-2, -0.08, 0.16	3, 0.18, 0.00	3, 0.11, 0.07	3, 0.27, 0.00	0.12
Land_TrN	-1, -0.17, 0.00	0, -0.24, 0.00	-2, -0.21, 0.00	-1, -0.28, 0.00	-0.23
Land_SH	-3, -0.18, 0.00	-3, -0.18, 0.00	3, -0.29, 0.00	3, -0.28, 0.00	-0.23
GlobalOcean	-1, -0.35, 0.00	1, -0.33, 0.00	1, -0.26, 0.00	1, -0.24, 0.00	-0.29
Ocean_NHL	3, -0.31, 0.00	3, -0.34, 0.00	3, -0.36, 0.00	3, -0.37, 0.00	-0.35
Ocean_MHL	-2, -0.22, 0.00	3, -0.28, 0.00	3, -0.23, 0.00	3, -0.25, 0.00	-0.25
Ocean_TrN	0, -0.15, 0.01	2, -0.13, 0.03	1, -0.04, 0.52	3, -0.06, 0.28	-0.10
Ocean_SH	0, -0.31, 0.00	0, -0.26, 0.00	0, -0.19, 0.00	1, -0.16, 0.01	-0.23
